# A Hierarchical Inverse Lithography Method Considering the Optimization and Manufacturability Limit by Gradient Descent

**DOI:** 10.3390/mi16070798

**Published:** 2025-07-08

**Authors:** Haifeng Sun, Qingyan Zhang, Jie Zhou, Jianwen Gong, Chuan Jin, Ji Zhou, Junbo Liu

**Affiliations:** 1 National Key Laboratory of Optical Field Manipulation Science and Technology, Chinese Academy of Sciences, Chengdu 610209, China; zhangqingyan22@mails.ucas.ac.cn (Q.Z.); zhoujie23@mails.ucas.ac.cn (J.Z.); gongjw@ioe.ac.cn (J.G.); jinchuan17@mails.ucas.ac.cn (C.J.); zhouji@ioe.ac.cn (J.Z.); ljbopt@126.com (J.L.); 2 State Key Lab of Optical Technologies on Nano-Fabrication and Micro-Engineering, Chinese Academy of Sciences, Chengdu 610209, China; 3 Institute of Optics and Electronics, Chinese Academy of Sciences, Chengdu 610209, China; 4 University of Chinese Academy of Sciences, Beijing 100049, China

**Keywords:** inverse lithography technology, gradient descent algorithm, resolution layering method

## Abstract

Inverse lithography technology (ILT) based on the gradient descent (GD) algorithm, which is a classical local optimal method, can effectively improve the lithographic imaging fidelity. However, due to the low-pass filtering effect of the lithography imaging system, GD, although able to converge quickly, is prone to fall into the local optimum for the information in the corner region of complex patterns. Considering the high-frequency information of the corner region during the optimization process, this paper proposes a resolution layering method to improve the efficiency of GD-based ILT algorithms. A corner-rounding-inspired target retargeting strategy is used to compensate for the over-optimization defect of GD for inversely optimizing the complex pattern layout. Furthermore, for ensuring the manufacturability of masks, differentiable top-hat and bottom-hat operations are employed to improve the optimization efficiency of the proposed method. To confirm the superiority of the proposed method, multiple optimization methods of ILT were compared. Numerical experiments show that the proposed method has higher optimization efficiency and effectively avoids the over-optimization.

## 1. Introduction

Lithography is a cornerstone of modern fabrication, extending far beyond integrated circuit (IC) manufacturing to critical applications in photonics and optoelectronics [1,2,3,4,5]. This versatile technique plays a pivotal role in the development of devices across various cutting-edge fields, including optical communication systems, metasurfaces, metalenses for light manipulation, optical micro-electromechanical systems (MEMSs) for sensing and actuation, and quantum photonics for quantum information processing. These applications rely on the precision of lithographic patterning to achieve the required miniaturization, accuracy, and functionality. Beyond ICs, lithography has become indispensable in producing intricate structures that manipulate light on both macroscopic and microscopic scales, driving advancements in technologies such as high-speed communication, imaging, and quantum computing. As a mainstream method of lithography, projection lithography technology employs a high-uniformity illumination system and a high-resolution objective system to implement the transfer of nanoscale pattern on the wafer surface, which is similar to the camera principle [6]. As the critical dimension (CD) of IC pattern continues to shrink, the numerical aperture (NA) of the objective system needs to be enlarged to ensure resolution at a constant illumination wavelength. Due to the objective lens system being a strictly diffraction-limited system, it is prone to the loss of high-frequency diffraction information during the imaging process, resulting in imaging distortion. The above phenomenon, known as the optical proximity effect (OPE) [7], will seriously affect the yield of IC manufacturing in the lithography process. Optical proximity correction (OPC), a resolution enhancement technique (RET), can effectively improve the imaging fidelity of lithography optical system by modifying the edges of feature patterns or adding assist features.

Rule-based OPC, as one of the traditional resolution enhancement techniques (RETs), is employed to eliminate the OPE in the early IC manufacturing process [8]. In this method, the correction rules are formed by calculating the offsets that need to be corrected for feature patterns under different conditions based on aerial image simulation or the actual pattern on the wafer surface. However, this method still has limitations for dealing with complex patterns [9]. In order to achieve higher resolution and imaging fidelity, the model-based OPC approach has been proposed, which centers on building a simulation system based on an optical model and a photoresist model to compute the desired lithographic feature pattern [10,11,12,13]. Nevertheless, due to the adaptability of the local correction scheme and Manhattan geometry correction constraints, the model-based OPC method is still limited under dense layouts [14].

Inverse lithography technology (ILT) with the model-based OPC approach as a starting point, as a core technology to enhance the lithographic imaging fidelity and process window in the advanced node, pixelates the feature pattern and optimizes the feature pattern layout via an optimization algorithm in reverse [15]. Gradient-based optimization algorithms, as a classical local optimization method, such as gradient descent (GD), conjugate gradient (CG), and steepest descent (SD), are widely employed to optimize the feature pattern layout in ILT for improving the lithographic imaging fidelity and limit resolution, which has the advantages of fast convergence and simple structure [16,17,18,19,20,21,22,23,24,25]. Xion et al. derived a gradient-based framework to automatically generate the optimum masks, which can reduce the edge placement errors and provide a good image fidelity [18]. Wei et al. proposed a multi-objective optimization with a defocus sensitivity penalty function as an objective to improve the lithographic process window via the mini-batch GD method [23]. For promoting the focusing performance, their method has been verified to be effective. Yu et al. employed a generic GD approach to optimize the lithographic imaging fidelity with two objective functions that are the resist and aerial image, respectively [17]. They confirmed that the combination of two objective functions in ILT based on GD can achieve a good feature pattern correction. These research results have confirmed that the gradient-based algorithm with multiple optimization strategies can match well with the lithographic imaging model and improve the imaging fidelity and process window. However, most of the objective functions in the above methods, such as aerial image error, resist image error, and edge placement errors (EPEs), were only considered to optimize a large range of errors between the actual simulation and the desired value for aerial images or resist patterns. The loss of high-frequency information, such as edges and corners of feature patterns, which is introduced by diffraction-limited factors, still cannot be rigorously addressed.

For optimizing the imaging fidelity utilizing ILT, in addition to the gradient-based optimization methods, researchers have employed other algorithms such as deep learning, machine learning, and heuristic algorithms with effective results. However, most of these methods are still focused around reducing the pattern errors and improving the match with lithographic imaging models. Considering that the gradient-based optimization method is prone to fall into a local optimum in the optimization of complex feature patterns, this paper aims to improve the optimization efficiency of GD-based ILT, reduce feature pattern manufacturability, and address the optimization of edges and corners information for aerial images. In this paper, a GD-based hierarchical inverse lithography method is proposed to resolve the above issues. For solving the over-optimization during the computation of ILT, a corner-rounding-inspired target retargeting strategy is employed to improve the optimized directionality. By incorporating differentiable top-hat and bottom-hat transformations—which detect feature size and spacing violations, respectively—our method explicitly trades off lithographic pattern fidelity against mask manufacturability constraints during gradient-based optimization. The experimental results show that the proposed method, compared with other ILT methods, improves the optimization efficiency by introducing a corner-rounding-inspired target retargeting strategy for optimizing imaging fidelity and manufacturability, which provides a superior solution for complex feature pattern optimization in advance nodes.

## 2. Lithography Imaging Model

The imaging process of the lithography system is based on the partially coherent theory, which is derived by the accumulation of a series of aerial images achieved by sub-light sources [9]. The imaging process of a point source is the same as completely coherent imaging, which can be expressed by Equations (1) and (2). In this completely coherent imaging model, it is assumed that a designed mask with the feature pattern can be represented as $m\in R^{N\times N}$.

(1) $$U_{S_{i}}\left(x,y;S_{i,x},S_{i,y}\right)=\iint H\left(f_{x}+S_{i,x},f_{y}+S_{i,y}\right)M\left(f_{x},f_{y}\right)e^{i2\pi \left[\left(f_{x}+S_{i,x}\right)x+\left(f_{y}+S_{i,y}\right)y\right]}df_{x}df_{y}$$

(2) $$I\left(x,y\right)=\iint S\left(S_{i,x},S_{i,y}\right){\left|U_{S_{i}}\left(x,y;S_{i,x},S_{i,y}\right)\right|}^{2}dS_{i,x}dS_{i,y}$$

where $U_{S_{i}}\left(x,y;S_{i,x},S_{i,y}\right)$ explains that the amplitude distribution of image generated by the $i_{th}$ sub-light source $S_{i}$ with coordinates $\left(S_{i,x},S_{i,y}\right)$ in the source of the partially coherent imaging model. $\left(f_{x},f_{y}\right)$ and $\left(f_{x}^{\prime },f_{y}^{\prime }\right)$ both express the spatial frequency coordinates on the pupil plane, $M\left(f_{x},f_{y}\right)$ represents the spectral information generated by the Fourier Transform of mask m, and $H\left(f_{x},f_{y}\right)$ denotes the projection pupil. Therefore, the intensity distribution $I\left(x,y\right)$ in the partially coherent imaging model can be expressed by Hopkins’ mode.

(3) $$I\left(x,y\right)=\iint \iint TCC\left(f_{x},f_{y},f_{x}^{\prime },f_{y}^{\prime }\right)M\left(f_{x},f_{y}\right)M^{*}\left(f_{x}^{\prime },f_{y}^{\prime }\right)e^{i2\pi \left[\left(f_{x}-f_{x}^{\prime }\right)x+\left(f_{y}-f_{y}^{\prime }\right)y\right]}df_{x}df_{y}df_{x}^{\prime }df_{y}^{\prime }$$

And

(4) $$TCC\left(f_{x},f_{y},f_{x}^{\prime },f_{y}^{\prime }\right)=\iint S\left(S_{i,x},S_{i,y}\right)H\left(f_{x}+S_{i,x},f_{y}+S_{i,y}\right)H^{*}\left(f_{x}^{\prime }+S_{i,x},f_{y}^{\prime }+S_{i,y}\right)dS_{i,x}dS_{i,y}$$

where $M^{*}\left(f_{x}^{\prime },f_{y}^{\prime }\right)$ and $H^{*}\left(f_{x}^{\prime },f_{y}^{\prime }\right)$ indicate the complex conjugate of M and H, respectively. The symbol S indicates the partial coherent source in the imaging. $TCC\left(f_{x},f_{y},f_{x}^{\prime },f_{y}^{\prime }\right)$ is the transmission cross-coefficient matrix. In order to reduce the computational complexity, the TCC matrix can be decomposed into a series of low-rank kernels by singular value decomposition. These low-rank kernels are explained as $h_{k}\in R^{P\times P}$, and each kernel is associated with an eigenvalue $\gamma_{k}$. Thus, the transformation from the feature patter of mask to aerial image can be approximated by Hopkins mode as shown in Equation (5).

(5) $$I\left(x,y\right)\approx \sum_{k=1}^{N}\gamma_{k}{\left|h_{k}\left(x,y\right)⊗m\left(x,y\right)\right|}^{2}$$

The method proposed is designed to solve the discrepancy of pattern layout between reality and ideal on the photoresist by optimizing the feature patterns in this paper. Moreover, the photoresist pattern (PRP) can be calculated by the resist effect approximated as the sigmoid function, which can be expressed as follows:

(6) $$I_{PRP}\left(x,y\right)=\Gamma \left[I\left(x,y\right)\right]=\frac{1}{1+\exp\left[-\alpha_{r}\left(I\left(x,y\right)-t_{r}\right)\right]}$$

Here, $I_{PRP}$ represents the intensity distribution of the PRP layout. The operational symbol $\Gamma \left[\cdot \right]$ is the sigmoid function, which is a regular S-type function. $\alpha_{r}$ is the steepness index, and $t_{r}$ is the threshold value of the photoresist.

## 3. Methodology

### 3.1. Inverse Lithography

The objective of ILT is to determine the optimal mask $m^{*}$ such that the resulting resist pattern closely matches the target design $I_{PRP}^{*}$. However, in practical fabrication processes, variations in process parameters such as defocus and exposure dose can cause deviations in the actual resist pattern [9]. To ensure performance stability under these variations, robustness must also be considered during optimization [17]. Mathematically, ILT can be formulated as an optimization problem in which the cost function $L_{c}$ is minimized to obtain a mask design that ensures high pattern fidelity and robust process performance, incorporating fidelity terms $L_{f}$ [26,27]. In addition, to ensure that the optimization process satisfies specific constraints, penalty terms $L_{r}$ are incorporated into the cost function to enforce design requirements and achieve a balanced trade-off.

(7) $$m^{*}=\underset{M}{arg\;min}\left(\beta_{f}L_{f}+\beta_{r}L_{r}\right)$$

where $L_{f}$ represents the fidelity metric and $L_{r}$ denotes the regularization penalty, each weighted by $\beta_{f}$ and $\beta_{r}$. The fidelity metric, quantified as the L2 loss, functions as a quantitative parameter for assessing the congruence between the intended target pattern and the actual resist pattern produced. It is mathematically represented by the squared norm of the element-wise difference between these two patterns [28].

(8) $$L_{f}=||I_{PRP}-I_{PRP}^{*}|{|}_{2}^{2}$$

Meanwhile, the regularization term $L_{r}$ introduces essential constraints that ensure the mask design remains manufacturable and adheres to practical fabrication requirements [29,30,31]. By incorporating both the fidelity metric and the regularization term into the optimization objective function, the method achieves a balanced trade-off between accurately reproducing the desired pattern and satisfying design constraints. Since a binary photomask can only represent two transmission levels—0 for opaque regions and 1 for transparent regions—directly optimizing these discrete states results in a challenging combinatorial problem that is computationally intractable [9]. To circumvent this difficulty, the mask design is relaxed into a continuous and differentiable pattern [26]. Specifically, the binary constraints are softened by mapping mask parameters onto a continuous interval between 0 and 1. This relaxation transforms the original discrete optimization into a continuous one, enabling the use of gradient-based optimization techniques and significantly improving computational efficiency [32].

(9) $$m\left(x,y\right)=\Gamma \left[m_{c}(x,y)\right]=\frac{1}{1+exp\left[-\alpha_{m}\left(m_{c}\left(x,y\right)-t_{m}\right)\right]}$$

Here, m refers to the binary mask, $m_{c}$ represents the continuous transmission mask (CTM), whose values are continuous and can vary within the interval [0, 1]. $\alpha_{m}$ and $t_{m}$ govern the sharpness and threshold of the sigmoid function, respectively [33]. Building upon these formulations, the modified fidelity cost function is derived and expressed as follows:


(10) $$L_{f}=\sum_{x=1}^{N}\sum_{y=1}^{N}{\left\{\frac{1}{1+\exp\left[-\alpha_{r}\left({\sum_{k=1}^{N}\gamma_{k}\left|\phi_{k}⊗\frac{1}{1+\exp\left[-\alpha_{m}\left(m_{c}(x,y)-t_{m}\right)\right]}\right|}^{2}-t_{r}\right)\right]}-R_{t}\right\}}^{2}$$

Drawing from the fidelity cost function above, the corresponding gradient of the CTM can be derived as follows [33,34]:

(11) $$\begin{array}{l} \nabla L_{f}=2\alpha_{r}\alpha_{m}m_{c}⊙\left(1-m_{c}\right)⊙\left[\Phi ⊙\left(I_{PRP}⊙\left(1-I_{PRP}\right)⊙\left(I_{PRP}-R_{t}\right)⊙\left(m_{c}⊙\Phi^{*}\right)\right)\right.\\ +\left.\Phi^{*}⊙\left(I_{PRP}⊙\left(1-I_{PRP}\right)⊙\left(I_{PRP}-R_{t}\right)⊙\left(m_{c}⊙\Phi \right)\right)\right] \end{array}$$

Here, Φ represents the sum of weighted convolution kernels, expressed as $\Phi =\sum_{k=1}^{K}\omega_{k}\phi_{k}$, and the circled dot symbol ⊙ denotes element-wise multiplication. The manufacturability metric consists of two components: the binary penalty term and the complexity penalty term [26]. The binary penalty term encourages the optimized mask pixels to be close to either 0 or 1, ensuring the mask adheres to the discrete nature of binary patterns. The complexity penalty term, on the other hand, prevents the mask from forming isolated or disconnected shapes, thereby promoting smooth and manufacturable designs. The gradient of the manufacturability metric can be expressed as $\nabla L_{r}$. Once the gradient is computed, the mask is updated iteratively using the gradient descent algorithm, as shown in the following update rule:

(12) $$m_{c}^{k+1}=m_{c}^{k}-\eta \left(\beta_{f}\nabla L_{f}+\beta_{r}\nabla L_{r}\right)$$

where $m_{c}^{i}$ denotes the optimized CTM obtained at the i-th iteration, and η represents the learning rate, which governs the step size during each optimization update. The gradient descent process continues iteratively until either the iteration count reaches a predefined maximum number of iterations or the cost function value decreases below a specified tolerance threshold. Once these stopping criteria are met, the optimization process concludes, yielding the resultant optimized CTM. Subsequently, a binarization step is applied to the optimized CTM to generate the corresponding binary mask $m_{final}$, which is defined as follows:

(13) $$m_{final}=\left\{\begin{array}{ll} 1, & M_{c}\geq 0.5\\ 0, & M_{c}<0.5 \end{array}\right.$$

**Figure 1:**
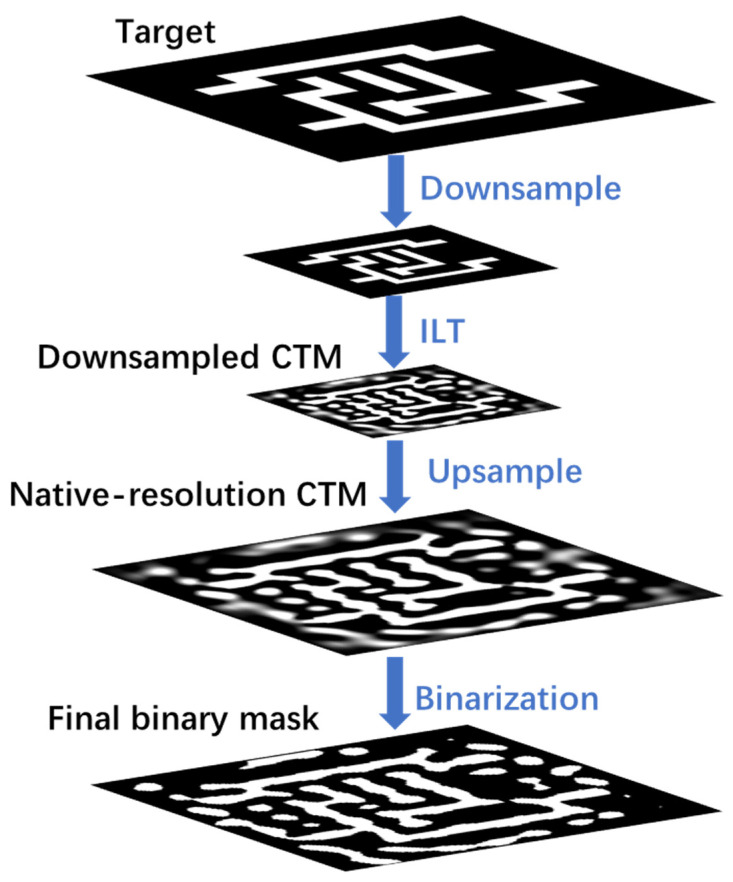
Hierarchical ILT optimization flowchart.

To enhance the efficiency of gradient-based ILT optimization, a hierarchical approach is employed by first performing optimization at a reduced spatial resolution. This reduction leads to a decrease in the computational burden of the optimization process [35,36]. When the mask downsampling factor is set to *s*, the downsampled mask is represented as m(sx,sy). Performing the ILT optimization on this lower-resolution mask results in faster convergence, as the optimization is conducted in a lower-dimensional parameter space. After the low-resolution mask is optimized, it is upsampled using interpolation techniques to obtain the high-resolution approximation optimization results. Finally, binarization is performed to generate the optimized binary mask. The schematic diagram of mask downsampling optimization is illustrated in Figure 1 below.

### 3.2. Corner Rounding

In edge-based OPC, the semiconductor industry typically adjusts the placement of edge placement error (EPE) measurement points to guide the OPC workflow more effectively. Instead of directly positioning these measurement points near polygon corners, which can lead to over-optimization, they are strategically relocated to the interior of convex corners or the exterior of concave corners [37,38]. This adjustment facilitates more efficient alignment of contours with the target during OPC, thereby reducing the risk of over-optimization and expanding the process window.

**Figure 2:**
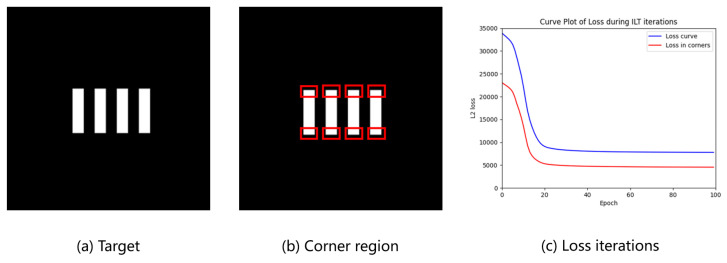
(**a**) The target design, (**b**) Corner region marked in red, and (**c**) their L2 loss iterations in ILT without corner rounding.

While ILT aims to optimize the entire design pattern rather than sampled control points, discrepancies between the resist and the design can still induce significant gradient variations during optimization. As illustrated in Figure 2, the objective function evolves throughout the optimization process. In the later stages of training, it becomes evident that mismatches at polygon corner regions continue to contribute substantially to the total cost. This persistent contribution often leads to premature convergence, hindering the achievement of further optimization improvements. As a result, the optimizer may disproportionately focus on infeasible targets, leading to suboptimal solutions that adversely affect the overall process robustness. Furthermore, conventional approaches that relocate EPE measurement points to mitigate corner-related challenges are inherently incompatible with pixel-based inverse optimization techniques [28].

The projection lithography system is diffraction-limited. Referring to the theorem of incoherent imaging, the cutoff frequency denoted as $f_{cut}$ is [35]

(14) $$f_{cut}=\frac{NA\left(1+\sigma_{out}\right)}{\lambda_{0}}$$

where *NA* denotes the numerical aperture, $\sigma_{out}$ represents the maximum partial coherence factor, and $\lambda_{0}$ corresponds to the illumination wavelength. So that we can estimate the limit of lithography imaging by the following equation [27].

(15) $$m_{filter}\left(x,y\right)=m\left(x,y\right)⊗K_{gauss}\left(x,y\right)$$

(16) $$K_{gauss}\left(u,v\right)=\frac{1}{2\pi \sigma^{2}}e^{-\frac{u^{2}+v^{2}}{2\sigma^{2}}},\left(\sigma =f_{cut}\right)$$

To approximate the ideal imaging characteristics of a lithographic system, the mask pattern is initially filtered using a Gaussian kernel to produce a grayscale mask representation. While this result does not represent the optimal lithographic output, the low-pass filtering properties effectively simulate the imaging behavior under idealized process conditions. In traditional ILT frameworks, the target pattern is inherently binary. To align the filtered grayscale mask with this binary requirement, thresholding is typically applied. However, arbitrary threshold selection can significantly influence the fidelity of the binarized output. This method introduces an edge-driven thresholding strategy: the edges of the original target pattern are first sampled, and the corresponding maximum pixel intensity values in the filtered mask are extracted as the threshold. Edge detection is implemented through morphological erosion operations, mathematically expressed as [39]:

(17) $$Erode\left(m,K\right)\left(i,j\right)=min\left\{m\left(i-k_{1},j-k_{2}\right)|\left(k_{1},k_{2}\right)\in K\right\}$$

where *K* is a 3 × 3 structural kernel during erosion. The disappearing region after erosion is the pattern contour.

(18) $$contour=m\left(i,j\right)-Erode\left(m,K\right)\left(i,j\right)$$

After selecting a threshold and binarizing, the mask can be retargeted to a corner rounding structure.

(19) $$Threshold=max\{m_{filtered}(i,j)⊙contour(i,j)\}$$

(20) $$m_{re}(x,y)=\left\{\begin{array}{cc} 0 & m\leq Threshold\\ 1 & m>Threshold \end{array}\right.$$

**Figure 3:**
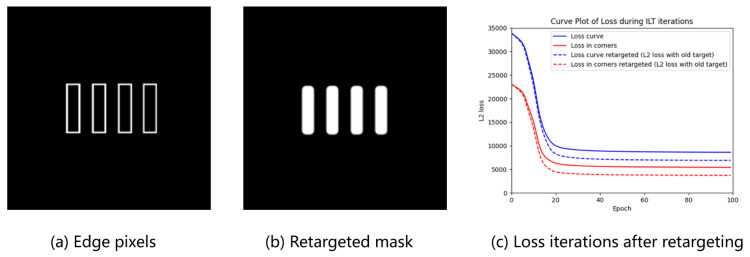
(**a**) The edge pixels, (**b**) the retargeted mask, and (**c**) comparison of their L2 loss iterations.

Figure 3 illustrates the re-targeting process in our framework: (a) depicts the extracted boundary elements from the original design, while (b) presents the realigned optimization target after applying our method. Notably, the sharp line ends in the raster structure are visibly smoothed post-realignment, indicating improved compatibility with lithographic resolution limits. The evolution of the cost function during optimization—both globally and specifically for corner regions—demonstrates that the proposed approach converges to a more robust solution. Compared to conventional methods, our strategy allocates reduced emphasis on non-optimizable edge features, thereby mitigating over-optimization associated with sub-wavelength pattern discontinuities.

### 3.3. MRC and Violation Penalty

The efficacy of ILT hinges not only on its ability to achieve high-fidelity wafer patterns but also on the practical feasibility of the mask itself. This is where mask rule checks (MRCs) become indispensable. MRCs refers to a set of geometric constraints applied to mask designs to ensure their manufacturability. These constraints typically include parameters such as [40,41]:

- Minimum feature size: The smallest allowable dimension of a pattern element;
- Minimum spacing: The smallest permitted distance between adjacent features;
- Minimum feature area: The smallest area allowed for a single feature.

**Figure 4:**
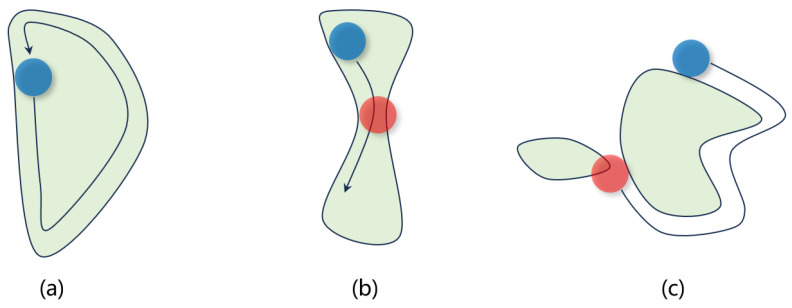
Examples of mask rule checks (MRCs) for a curvilinear mask. (**a**) The minimum circle can slide entirely within and around the mask, so that it is MRC-clean. (**b**) The internal checking fails, and it violates the minimum feature size setting. (**c**) The external checking fails, and it violates the minimum spacing setting.

Without considering MRCs, the optimized masks cannot be applied for manufacturability. Compared with Manhattan-shaped masks, the task of MRCs is simpler. The MRC involves two steps. First, a circle of a specific diameter (blue in Figure 4) is rolled along the interior of the mask shape. This ensures the circle remains entirely within the mask structure during movement. Second, a second circle with a potentially different diameter (red in Figure 4) is rolled along the exterior of the shape. This guarantees the circle stays outside the mask region and does not encroach on adjacent features. Both operations require the circles to traverse the contour completely without violating their respective spatial constraints.

Integrating MRCs into the ILT workflow is critical for ensuring manufacturability. Compared to post-optimization MRC correction, a differentiable quantitative metric that evaluates the degree of design rule violations and integrates it into the optimization cost function enables graded penalties for non-compliant patterns. This approach enhances the ability of ILT to generate manufacturable masks while improving pattern fidelity. The morphological operations described above—such as rolling a circle along the interior and exterior contours of a design—can be mathematically represented through morphological processing [20]. Originally introduced in the 1980s for image processing [42,43], morphological operations include fundamental operations like dilation (expanding patterns by merging features within a kernel radius) and erosion (removing structures smaller than the kernel). Advanced operations such as opening (erosion followed by dilation) and closing (dilation followed by erosion), as well as top-hat and bottom-hat transformations, further refine feature extraction. Specifically, the top-hat operation highlights small-scale structures smaller than the kernel, while the bottom-hat operation identifies narrow gaps. In our proposed framework, we achieve a novel integration by applying differentiable top-hat and bottom-hat operations for the first time to characterize minimum feature size and spacing violations in MRC. These operations are explicitly formulated as penalty terms in the optimization cost function to enforce manufacturability constraints.

**Figure 5:**
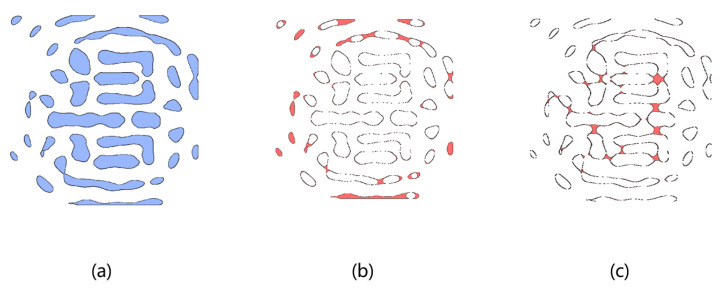
MRC violation analysis: (**a**) traditional ILT mask; (**b**) feature width violations (red regions); (**c**) spacing violations (red regions).

As shown in Figure 5a, this example illustrates a mask design generated by conventional ILT without MRC integration, where blue regions represent the optimized mask pattern. Figure 5b highlights red regions identified as MRC violations through the application of a top-hat operation, while Figure 5c demonstrates the detection of spacing violations (red regions) using a bottom-hat operation. It is important to note that traditional morphological operations are inherently non-differentiable. However, in our proposed framework, gradient-based optimization algorithms require differentiable components to compute gradients and update the mask iteratively. To address this requirement, we implement a differentiable morphological computation framework utilizing pooling operations and Gaussian approximation techniques. Specifically, we employ maximum pooling operations to approximate dilation processes and minimum pooling operations to simulate erosion effects, both configured with appropriate structural kernels. This approach ensures continuous gradient propagation during the optimization process, enabling effective detection of mask rule check (MRC) violations—such as sub-resolution features or insufficient spacing—and their subsequent penalization within the composite loss function. The specific implementation details of these operations are summarized in Algorithms 1 and 2 below:
**Algorithm 1:** Differentiable morphological operation1:**function** Differentiable_Dilation(**m**∈ℝ^N×N^, **K**∈ℝ^r×r^)2:**m^+^**←ZeroPadding(M, ⌊r/2⌋)3:**m**(i,j)←max(**m^+^**(i:i+k,j:j+k)⊙**K**)4:**return m**5:**function** Differentiable_Erosion(**m**∈ℝ^N×N^, **K**∈ℝ^r×r^)6:**m^+^**←ZeroPadding(M, ⌊r/2⌋)7:**m**(i, j)←min(**m^+^**(i: i+k, j: j+k)⊙**K**)8:**return m**9:**function** Differentiable_Opening(**m**∈ℝ^N×N^, **K**∈ℝ^r×r^)10:**m**←Differentiable_Dilation(Differentiable_Erosion(**A**,**K**),**K**)11:**return m**12:**function** Differentiable_Closing(**m**∈ℝ^N×N^, **K**∈ℝ^r×r^)13:**m**←Differentiable_Erosion(Differentiable_Dilation(**A**,**K**),**K**)14:**return m**

**Algorithm 2:** Differentiable manufacturability penalty
1:**function** Feature_violation_penalty(**m**∈ℝ^N×N^, **K**∈ℝ^r×r^)2:**m_v1_**←m − Differentiable_Opening(m, K)3:**P**←sum(**m_v1_(:,:)**)4:
**return P**
5:**function** Space_violation_penalty(**m**∈ℝ^N×N^, **K**∈ℝ^r×r^)6:**m_v2_**←Differentiable_Closing(m, K)-m7:**P**←sum(**m_v2_(:,:)**)8:
**return P**



## 4. Experiments and Results

Our proposed algorithm is implemented in Python 3.8 with PyTorch 2.0. The simulation experiments are conducted on a computer with an NVIDIA GeForce 4090 GPU and a 3.8 GHz AMD Ryzen CPU. To analyze the performance of our proposed method, we utilize the ICCAD13 CAD Contest benchmark to carry out ablation studies. The ICCAD13 benchmark consists of ten 2 μm × 2 μm M1 metal layers. We compare the results of other State-of-the-Art works to justify our method’s advantages.

**Figure 6:**
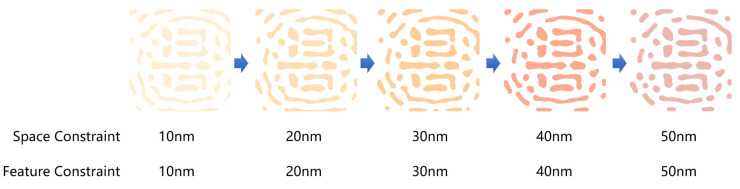
The optimized masks by different space and feature width constraints.

To validate the effectiveness of the proposed MRC penalty function introduced in this paper, we examined the layout optimization results from ICCAD13 under varying MRC constraints. The evolution of these results, as illustrated in the figures, demonstrates that our method significantly enhances the optimization reliability across different mask manufacturing conditions. As shown in the Figure 6, when space constraints and feature size constraints are varied from 10 nm to 60 nm, the resulting optimized masks exhibit notable differences, particularly in the Sub-Resolution Assist Features (SRAFs). With stricter space and feature constraints, the SRAFs demonstrate increased spacing and linewidths, aligning with the specified MRC requirements. These findings confirm that our approach effectively accommodates a range of manufacturing constraints while maintaining high fidelity and manufacturability.

**Figure 7:**
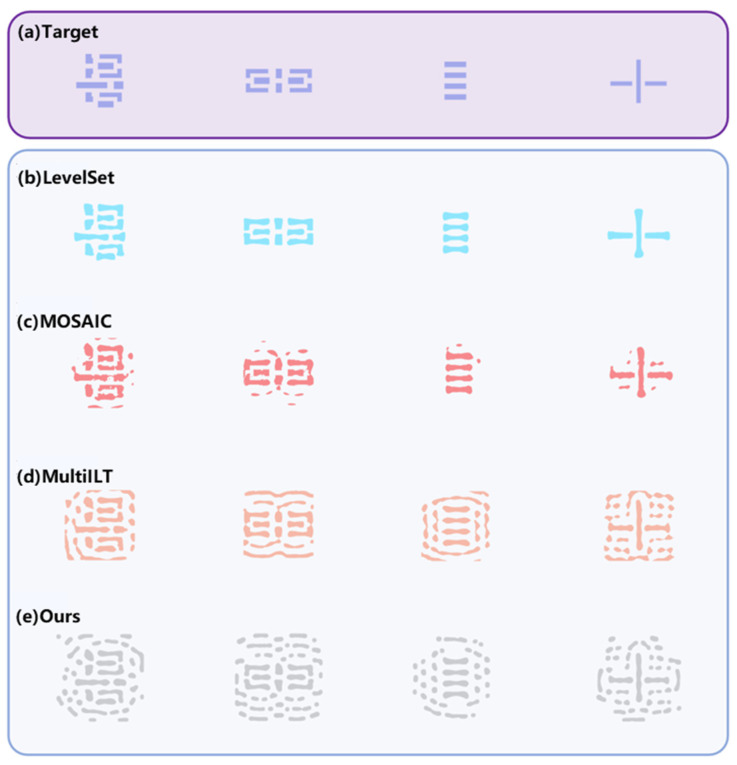
Simulation results for different patterns. (**a**) Target; (**b**) LevelSet; (**c**) MOSAIC; (**d**) MultiILT; (**e**) ours.

In the comparative experiment, our method employs a spacing constraint of 10 nm and a feature size (linewidth) constraint of 40 nm. Figure 7 presents the results of optimized mask patterns for various methods on complex layouts. The top row shows the target pattern used in ILT, while the subsequent rows depict the optimized masks obtained by LevelSet [44], MOSAIC [45], MultiILT [46], and our proposed method, respectively. Visually, LevelSet only optimizes the main features without generating any Sub-Resolution Assist Features (SRAFs). The MOSAIC method introduces some SRAFs, but they appear irregular and lack manufacturability. In contrast, MultiILT, which adopts a hierarchical optimization strategy, achieves improved pattern fidelity and enhanced manufacturability. However, it still produces certain features that violate MRC rules.

Compared to these existing approaches, our proposed method demonstrates a clear improvement in manufacturability. The generated SRAFs are well-structured and fully compliant with the specified MRC requirements.

From the metrics presented in Table 1, the superiority of our proposed method becomes more evident. Before delving into the comparisons, let us first define the metrics used:

- MSE: Represents the L2 loss as defined in Equation (3);
- PVB (Process Variation Bandwidth): Characterizes manufacturing process robustness by calculating the maximum contour separation area between the outermost contour $I_{PRP,out}$ and the innermost contour $I_{PRP,in}$ under various process conditions;
  (21) $$PVB=||I_{PRP,out}-I_{PRP,in}|{|}_{2}^{2}$$
- EPE: Measures the critical dimension at predefined edge test positions. If it exceeds a specified threshold, it is counted as an error;
- For manufacturability, two metrics are employed: FVP (Feature Violation Penalty) and SVP (Space Violation Penalty), corresponding to the space constraint penalty and feature constraint penalty described in Algorithm 2.

Given that the LevelSet and MOSAIC methods exhibit poor manufacturability, their FVP and SVP metrics are not analyzed here. Overall, our proposed method demonstrates superior performance across all metrics. Specifically, in terms of MSE, our method shows an average improvement of approximately 6.2% over the next best method, MultiILT, while maintaining a comparable PVB. Regarding manufacturability, our method achieves FVP and SVP values that are 3.7% and 4.5% better than those of MultiILT, respectively. This indicates that our approach successfully integrates MRCs into ILT, thereby enhancing the manufacturability of the optimized masks.

## 5. Conclusions

In this work, we propose a hierarchical pixel-based inverse lithography technology framework, addressing the current challenges and advancements in ILT algorithm development. By considering mask optimization with manufacturability constraints and optimization limits, we introduce two key innovations. First, a corner-rounding-inspired target retargeting strategy is developed to mitigate over-optimization during the ILT process. This approach significantly enhances both convergence efficiency and final result quality, as evidenced by experimental results showing a 17% improvement in post-convergence performance for the grating pattern. For complex patterns, it improves by 6.2% averagely. Second, we incorporate MRC requirements into the ILT workflow through differentiable top-hat and bottom-hat operations. This ensures a balanced trade-off between pattern fidelity and manufacturability while enabling flexibility to generate solutions tailored to specific MRC complexity levels. However, the current manufacturability cost function does not account for full-chip optimization scenarios, particularly MRC violations at tile boundaries. Future work will focus on extending the framework to address these cross-tile manufacturability challenges, thereby improving scalability and robustness in large-scale photomask design.

## Figures and Tables

**Table 1:** Comparison of ICCAD13 results with other ILT methods.

Case	LevelSet			MOSAIC			MultiILT					Ours				
	MSE	PV	EPE	MSE	PV	EPE	MSE	PV	EPE	FVP	SVP	MSE	PV	EPE	FVP	SVP
1	45,520	57,468	6	48,896	55,028	8	39,533	44,887	3	2282	23	39,984	44,380	3	440	20
2	33,571	49,680	1	37,327	46,019	4	32,516	37,374	0	1102	50	29,962	36,806	0	110	31
3	78,695	90,748	39	81,327	86,685	47	65,315	75,011	23	4648	65	62,374	72,197	15	120	20
4	18,040	27,710	2	16,409	26,358	2	9099	21,484	0	3899	905	8648	22,746	0	99	25
5	38,226	59,035	2	37,810	57,472	0	30,015	48,696	0	2321	716	28,908	47,368	0	284	14
6	35,962	54,163	0	36,706	52,566	0	33,400	42,788	0	5917	351	29,844	42,230	0	71	26
7	30,542	48,173	2	29,520	47,598	2	17,419	36,241	0	3418	573	14,098	36,414	0	56	19
8	14,252	25,043	1	14,291	24,268	1	11,552	18,987	0	4922	1083	10,292	19,631	0	70	28
9	43,390	68,229	1	47,367	64,932	2	37,219	54,792	0	2777	707	34,435	54,336	0	69	16
10	8919	20,878	0	8950	19,871	0	7180	14,979	0	5270	382	7193	15,796	0	33	19
Avg	34,712	50,113	5.4	35,860	35,860	6.6	28,325	39,524	2.6	3656	486	**26,574**	**39,190**	**1.8**	**135**	**22**

## Data Availability

All data are included in the study.
